# A human induced pluripotent stem cell model from a patient with hereditary cerebral small vessel disease carrying a heterozygous R302Q mutation in *HTRA1*

**DOI:** 10.1186/s41232-023-00273-7

**Published:** 2023-04-03

**Authors:** Emi Qian, Masahiro Uemura, Hiroya Kobayashi, Shiho Nakamura, Fumiko Ozawa, Sho Yoshimatsu, Mitsuru Ishikawa, Osamu Onodera, Satoru Morimoto, Hideyuki Okano

**Affiliations:** 1 Department of Physiology, Keio University School of Medicine, Tokyo, Japan; 2 Department of Neurology, Brain Research Institute, Niigata University, Niigata, Japan

**Keywords:** Cerebral small vessel disease, hiPSC, Hereditary disease, *HTRA1*, CARASIL, Arteriopathy, Stroke

## Abstract

Cerebral autosomal recessive arteriopathy with subcortical infarcts and leukoencephalopathy (CARASIL) is an inherited cerebral small vessel disease (CSVD) caused by biallelic mutations in the *high-temperature requirement serine peptidase A1 (HTRA1)* gene. Even heterozygous mutations in *HTRA1* are recently revealed to cause cardinal clinical features of CSVD. Here, we report the first establishment of a human induced pluripotent stem cell (hiPSC) line from a patient with heterozygous *HTRA1*-related CSVD. Peripheral blood mononuclear cells (PBMCs) were reprogrammed by the transfection of episomal vectors encoding human *OCT3/4* (*POU5F1*), *SOX2*, *KLF4*, *L-MYC*, *LIN28*, and a murine dominant-negative mutant of *p53* (*mp53DD*). The established iPSCs had normal morphology as human pluripotent stem cells and normal karyotype (46XX). Moreover, we found that the *HTRA1* missense mutation (c.905G>A, p.R302Q) was heterozygous. These iPSCs expressed pluripotency-related markers and had the potential to differentiate into all three germ layers in vitro. *HTRA1* and the supposed disease-associated gene *NOG* were differentially expressed in the patient iPSCs at mRNA levels compared to those of control lines. The iPSC line would facilitate in vitro research for understanding the cellular pathomechanisms caused by the *HTRA1* mutation including its dominant-negative effect.

**Supplementary Information:**

The online version contains supplementary material available at 10.1186/s41232-023-00273-7.

## Background

Cerebral small vessel disease (CSVD) is a spectrum of clinical and imaging findings caused by the structural change of small arteries, arterioles, capillaries, and venules in the brain [1]. Neuroimaging exhibits white matter hyperintensities, lacunar infarcts, small subcortical infarcts, microbleeds, and enlarged perivascular spaces [1]. Clinical features of CSVD include stroke, cognitive dysfunction, dementia, psychiatric disorders, and gait abnormality [1]. Currently, immune responses are increasingly recognized as critical elements contributing to the development of CSVD [2]. The endothelial dysfunction and monocytes/macrophages activation is assumed to increase the permeability of the blood-brain barrier, leading to abnormal autoregulation of cerebral blood flow in CSVD [3, 4]. Growing evidence indicates the association of inflammation markers with CSVD [3–5]. As the pathomechanisms of CSVD are yet little known, the study of monogenic hereditary forms of CSVD is expected to be one of the effective approaches to revealing how cerebral lesions are formed in CSVD.

Cerebral autosomal recessive arteriopathy with subcortical infarcts and leukoencephalopathy (CARASIL) is a rare inherited CSVD with clinical manifestations of cerebral small vessel arteriopathy, severe leukoaraiosis, multiple lacunar infarcts, premature alopecia, and back pain/spinal degeneration at an earlier age. The cause was identified in Japan as recessive mutations in the *high-temperature requirement serine peptidase A1 (HTRA1)* gene [6], which encodes an ATP-independent serine protease preventing the accumulation and aggregation of misfolded proteins that may impose severe damage to cells. A previously proposed theory of disease pathology is that the consequent loss of function of *HTRA1* results in the disinhibition of the transforming growth factor β1 (TGF-β1) signal, which causes hair loss, bone formation abnormality, capillary dilation with hemorrhage, and vascular fibrosis [6]. A recent study using *Htra1*^−/−^ mice has disclosed that the loss of function of *Htra1* caused the pathological accumulation of matrisome proteins including fibronectin and latent TGF-β binding protein 4 (Ltbp4) in the thickened intima of cerebral large vessels, pial arteries, and arterioles, which might affect vascular distensibility and cerebral blood flow, leading the disease onset [7]. However, the detailed regulatory associations between *HTRA1* and TGF-β1 signal, or the comprehensive effects of accumulated proteins including matorisome and even non-matrisome proteins are not fully elucidated. Besides, why the expression of clinical phenotypes is localized to specific sites remains unclear.

Surprisingly, increasing evidence has shown that even heterozygous *HTRA1* mutations can contribute to symptomatic CSVD [8–10]. Some critical heterozygous mutation sites in *HTRA1* such as p.G283E, p.P285L, p.T319I, and p.R302Q present mild to severe symptoms of CARASIL [8]. Heterozygous *HTRA1*-related CSVD is likely to exhibit reduced HTRA1 protease activity as well as CARASIL since in vitro fluorogenic assay showed a mixture of wild-type and mutant HTRA1 reduced protease activity [8, 11]. A proposed possible pathomechanism is that the dominant-negative effect of mutant *HTRA1* leads to the deterioration of overall *HTRA1* protease activity through protein conformation defects [8, 11]. However, the precise mechanism by which the heterozygous mutation leads to apparent symptomatic CSVD needs further investigation.

To establish an in vitro human disease model, human induced pluripotent stem cell (hiPSC) has been a remarkable tool for elucidating cellular pathomechanisms and application to regenerative medicine [12–14]. The reprogramming method to establish iPSC from skin fibroblasts was first developed in mice [15] and then in humans [16] by the overexpression of reprogramming factors such as *OCT3/4*, *SOX2*, *KLF4*, and *c-MYC* using retrovirus infection. Eventually, transgene integration turned out to be unnecessary for maintaining pluripotency, leading to the development of methods to derivate integration-free iPSCs using oriP/EBNA1-based episomal vectors [17]. Additionally, since skin biopsies for hiPSC establishment are highly invasive and fibroblasts require several passages for expansion, novel efficient methods were searched to derivate integration-free hiPSCs from peripheral blood-derived T lymphocytes [18–20]. With these efforts to develop efficient and applicable methods to clinical contexts, hiPSCs are now widely used for disease modeling, drug discovery, and transplantation. Still, there is much more potential to utilize hiPSCs derived from various patients with diseases that are not fully understood in terms of disease pathology or effective treatments.

In this study, we generated a hiPSC line named SM9-1 (also named KEIOi003-A) from a 70-year-old female patient with *HTRA1*-related CSVD (heterozygous R302Q mutation in *HTRA1*). We used oriP/EBNA1-based episomal vectors for iPSC establishment. We performed validation for the established iPSC line and *HTRA1*-related gene expression analysis. Since this is the first reported hiPSC line derived from a patient with heterozygous *HTRA1*-related CSVD, it could be a powerful tool to address the undiscovered pathological mechanisms of hereditary CSVD by taking advantage of its pluripotency to differentiate into targeted tissues and organs.

## Main text

**Fig. 1:**
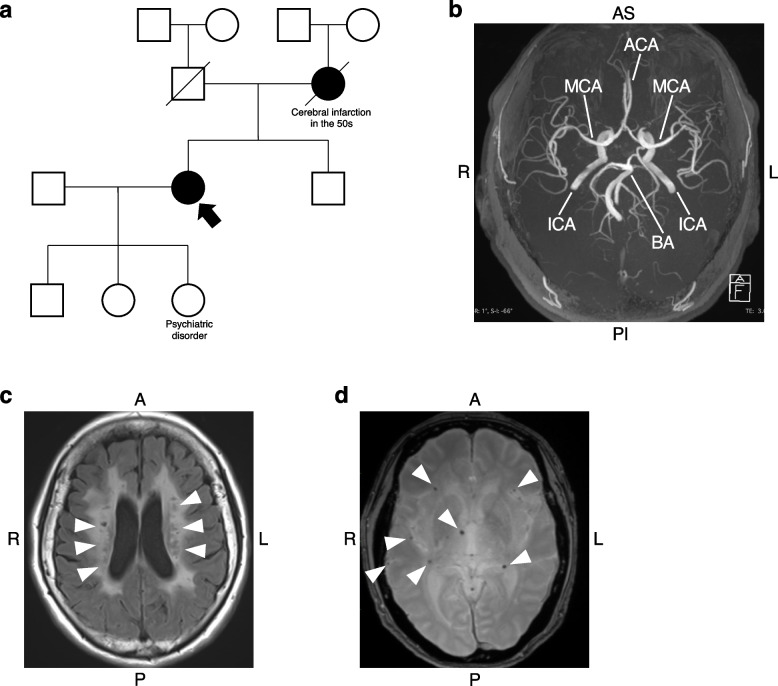
Clinical findings of the patient carrying the heterozygous *HTRA1* mutation.** a** Family pedigree of the patient with heterozygous *HTRA1*-related CSVD. Squares, men; circles, women; filled symbols, individuals with a history of a stroke; a filled arrow, the donor for iPSC establishment; diagonal lines through symbols, deceased members. **b** Image of magnetic resonance angiography (MRA) acquired at the age of 69. ACA; anterior cerebral artery, MCA; middle cerebral artery, ICA; internal carotid artery, BA; basilar artery, L, left; R, right; AP, anterior superior; PI, posterior inferior. **c**. Fluid-attenuated inversion recovery (FLAIR) image of the brain magnetic resonance imaging (MRI) acquired at the age of 69. Ischemic changes are indicated by arrowheads. L left; R, right; A, anterior; P, posterior. **d**. T2*-weighted Image (T2*WI) of the brain MRI acquired at the age of 69. Microbleeds are marked with arrowheads

We recruited a 70-year-old symptomatic female with heterozygous *HTRA1*-related CSVD and obtained consent to establish a hiPSC line (Table 1) [21]. The family pedigree shows a stroke history in her mother (Fig. 1a). One of her daughters may be a potential CSVD patient as she has a psychiatric disorder (one of the symptoms of CSVD). The family pedigree implies the possibility of hereditary CSVD with an autosomal dominant pattern of inheritance over 2 or 3 generations, which is consistent with heterozygous *HTRA1*-related CSVD. The patient’s first episode was a stroke at the age of 53. She was sequentially diagnosed with depression at the age of 63 and normal pressure hydrocephalus in the following year. Laminectomy and vertebroplasty were conducted for cervical spondylosis. Higher brain dysfunction, parkinsonism, hyperactive tendon reflexes, and pathologic reflexes (jaw jerk, Wartenberg, Trömner, Babinski, and Chaddock) were observed without alopecia or hypertension. Magnetic resonance angiography (MRA) showed no stenosis or aneurysm with the normal formation of large cerebral vessels (Fig. 1b). Brain MRI (Fluid-attenuated inversion recovery; FLAIR) revealed extensive ischemic changes in the bilateral cerebral white matter with old infarcts in the basal ganglia and left thalamus (Fig. 1c). T2* weighted Image (T2*WI) revealed diffused microbleeds (Fig. 1d). The patient carried a heterozygous missense mutation in exon 4 of *HTRA1*. The mild symptoms such as older-onset stroke and dementia, and no alopecia can be possibly attributed to the heterozygous mutation. The affected amino acid (R302Q) locates on a component of the L3 domain, which is essential for the *HTRA1* protease activities.

**Table 1:** Resource information of the established iPSC line

iPSC line name	KEIOi003-A (unique name at hPSCreg) SM9-1 (alternative name)
Institution	Keio University School of Medicine, Tokyo, Japan
Origin information	Species: human, Sex: female, Age: 70 years old, Ethnicity: Japanese Cell source: peripheral blood mononuclear cells
Clonality	Clonal
Method of reprogramming	Electroporation using the episomal vectors: pCE-hOCT3/4, pCE-hSK, pCE-hUL, pCE-mp53DD, and pCXB-EBNA1
Name of transgenes	*OCT3/4*, *SOX2*, *KLF4*, *L-MYC*, *LIN28*, mutant *Trp53* (removed after the establishment)
Genetic modification	Spontaneous mutation *HTRA1*, c.905G>A, p.R302Q, heterozygous
Associated disease	Heterozygous HTRA1-related cerebral small vessel disease
Summary of clinical characteristics	**History (age at onset):** Stroke (53 years old), depression (63 years old), normal pressure hydrocephalus (64 years old), and cervical spondylosis **Examination findings:** •No alopecia or hypertension •Higher brain dysfunction, parkinsonism, hyperactive tendon reflexes, pathologic reflexes, and cognitive function decline •The Japanese version of the Montreal Cognitive Assessment (MoCA-J) score: 12 points (71 years old) •Brain MRI: Ischemic changes in the bilateral cerebral white matter, old infarcts in the basal ganglia and left thalamus, diffused microbleeds (69 years old)
Donor screening for infections	Negative for human immunodeficiency virus (HIV) antibody, hepatitis B surface (HBs) antigen, hepatitis C virus (HCV) antibody, serological tests for syphilis (STS) and treponema pallidum hemagglutination test (TPHA)

**Fig. 2:**
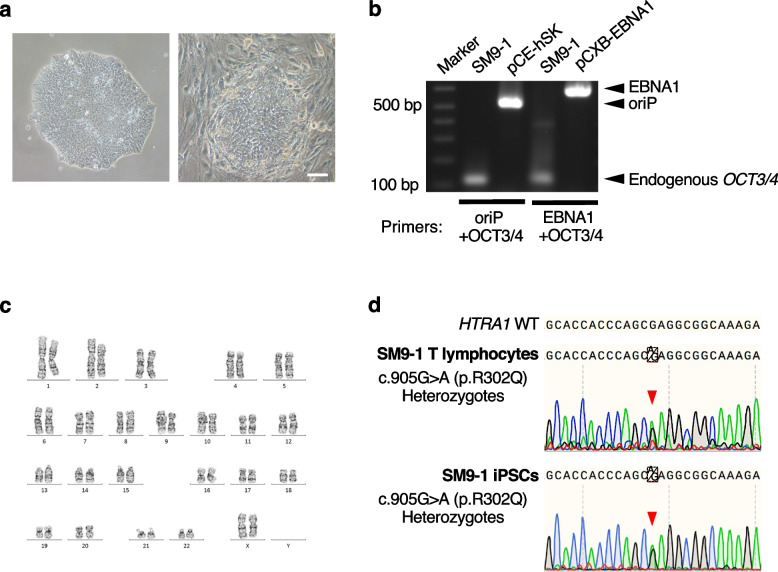
Characterization of SM9-1 iPSCs. **a** Morphology of the established iPSC line SM9-1 (left panel: feeder-free condition, right panel: on-feeder condition). Scale bar, 100 μm. **b** PCR analysis for the detection of remaining oriP/EBNA1-based reprogramming vectors. **c** Karyotype analysis of SM9-1 iPSCs. **d** Sanger sequencing of the targeted mutation site of *HTRA1* gene (c.905G>A, p. R302Q, heterozygous) in SM9-1 T lymphocytes and SM9-1 iPSCs

For iPSC establishment, peripheral blood mononuclear cells (PBMCs) from the patient were reprogrammed by electroporation of episomal vectors (pCE-hOCT3/4, pCE-hSK, pCE-hUL, pCE-mp53DD, and pCXB-EBNA1) [20], and cultured in feeder-free and on-feeder conditions. Putative iPSC colonies were manually picked up, one of which was named SM9-1. SM9-1 iPSCs exhibited standard stem cell-like morphology in feeder-free and on-feeder conditions (Fig. 2a). The remaining episomal vectors were not detected by genomic PCR at passage 5 (Fig. 2b). Also, SM9-1 iPSCs at passage 5 were used for karyotyping and showed a standard diploid 46, XX karyotype (Fig. 2c). Sanger sequencing detected the heterozygous missense mutation c.905G>A (p. R302Q) in exon 4 of *HTRA1* both in SM9-1 T lymphocytes and SM9-1 iPSCs at passage 5 (Fig. 2d). Short tandem repeat (STR) analysis was performed using SM9-1 T lymphocytes and SM9-1 iPSCs at passage 4. The examined 10 loci (TH01, D21S11, D5S818, D13S317, D7S820, D16S539, CSF1PO, AMEL, vWA, and TPOX) were identical (Supplementary Table S1). The mycoplasma contamination test was negative at passage 4 (Supplementary Table S2).

**Fig. 3:**
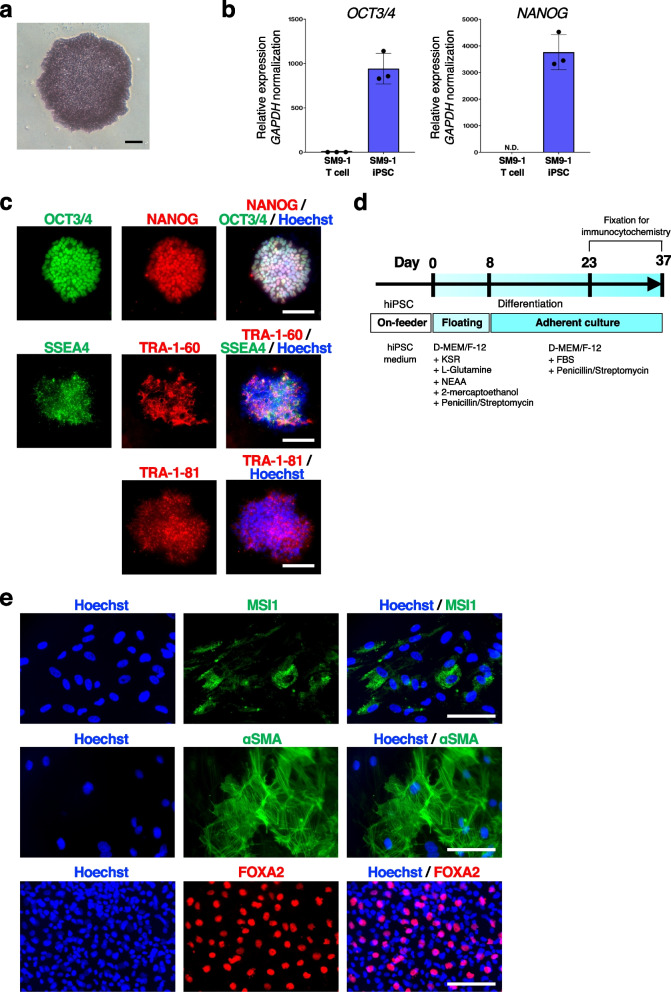
Pluripotency validation of SM9-1 iPSCs.** a** Alkaline phosphatase staining of an SM9-1 iPSC colony. Scale bar, 100 μm. **b** RT-qPCR analysis to examine the expression levels of pluripotency marker genes in SM9-1 T lymphocytes and SM9-1 iPSCs. Values represent the mean ± standard error of the mean (SEM) of technical triplicates. **c** Immunocytochemistry of human pluripotency markers. Scale bar, 100 μm. **d** Schematic summary of culture protocol for spontaneous differentiation. **e** Immunocytochemistry of three germ layers markers using spontaneously differentiated cells in vitro. Scale bar, 100 μm

We next validated the pluripotency of SM9-1 iPSCs. The iPSCs at passage 6 showed alkaline phosphatase activity (Fig. 3a). Reverse transcription-quantitative real-time PCR (RT-qPCR) exhibited strong expression of pluripotency markers such as *NANOG* and *OCT3/4* in SM9-1 iPSCs at passage 7 (Fig. 3b). Besides, SM9-1 iPSCs were immunopositive for human pluripotency markers such as TRA-1-60, TRA-1-81, SSEA4, NANOG, and OCT3/4 by immunocytochemistry (Fig. 3c). The potential to differentiate into all three germ layers was validated by in vitro spontaneous differentiation (Fig. 3d). Embryoid bodies (EBs) were successfully formed, and positive cells for ectoderm (MSI1), mesoderm (αSMA), and endoderm (FOXA2) markers were respectively found by immunocytochemistry (Fig. 3e).

**Fig. 4:**
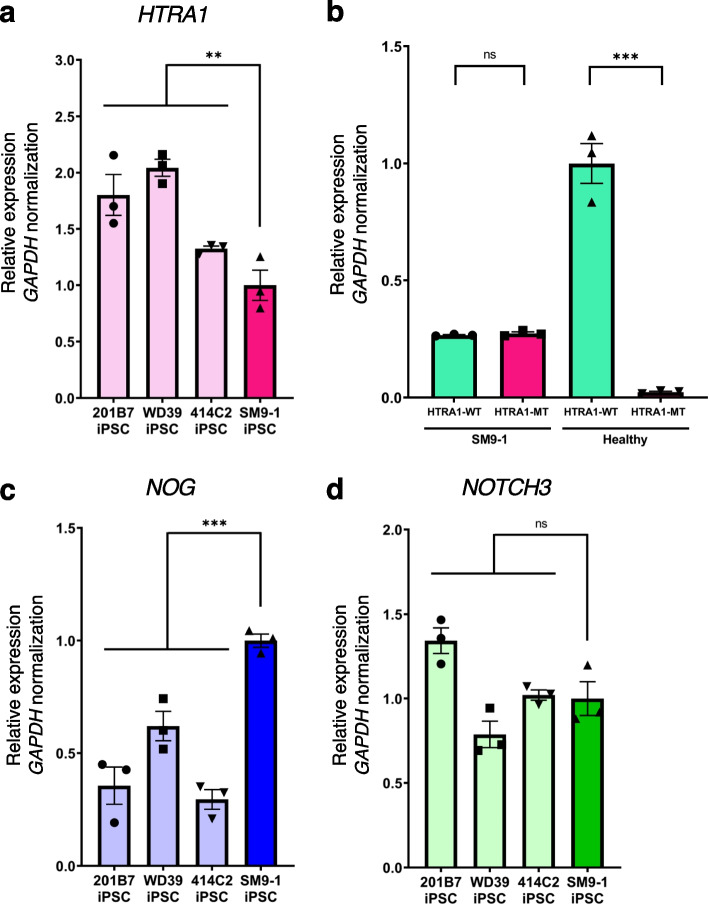
Gene expression profiling of SM9-1 iPSCs. **a** RT-qPCR analysis to examine the expression levels of *HTRA1* and associated genes in SM9-1 T lymphocytes and SM9-1 iPSCs compared with healthy iPSCs (201B7, WD39, and 414C2). Values represent the mean ± SEM of technical triplicates; **p < 0.01. **b** RT-qPCR analysis to compare the expression levels of wild-type and mutant *HTRA1* RNA in SM9-1 iPSCs and healthy iPSCs (201B7). Values represent the mean ± SEM of technical triplicates; ***p < 0.001. **c**,** d** RT-qPCR analysis to examine the expression levels of *NOG* and *NOTCH3* in SM9-1 T lymphocytes and SM9-1 iPSCs compared with healthy iPSCs (201B7, WD39, and 414C2). Values represent the mean ± SEM of technical triplicates; ***p < 0.001

Subsequently, we examined the mRNA expression levels of supposed disease-associated genes using SM9-1 iPSCs. RT-qPCR revealed that *HTRA1* expression decreased in SM9-1 iPSCs (Fig. 4a). A decrease in *HTRA1* mRNA expression has been reported in skin fibroblasts of a CARASIL patient carrying homozygous nonsense mutations (R370X) in *HTRA1*, although it is considered nonsense mutation-mediated RNA decay [6]. However, there was no significant downregulation in the expression level of mutant RNA compared to wild-type RNA in the patient-derived iPSC with missense mutation (R302Q) (Fig. 4b). On the other hand, overall *HTRA1* RNA levels were lower in patient-derived cells than in healthy controls, suggesting R302Q affects the overall decline of *HTRA1* RNA translation rather than mutant-specific RNA decay. We also revealed *NOGGIN* gene (*NOG*) expression, which is upregulated by TGF-β1 signaling in fibroblasts [22], was increased in SM9-1 iPSCs (Fig. 4c). There seems to be a small effect on its pluripotency caused by upregulated *NOG* expression at iPSC level as there was no significant difference in the expression of *NANOG* and *OCT3/4* between healthy iPSC lines and SM9-1 iPSC line (Supplementary Figure S1). Additionally, we evaluated *NOTCH3* expression in SM9-1 iPSCs but found no statistical difference in *NOTCH3* expression (Fig. 4d). *NOTCH3* is known as the causative gene of cerebral autosomal dominant arteriopathy with subcortical infarcts and leukoencephalopathy (CADASIL) [23]. CADASIL is also an inherited CSVD with similar clinical findings to heterozygous *HTRA1*-related CSVD and CARASIL. These CSVDs have been recognized as overlapping diseases with some shared molecular mechanisms. Brain vessels of a CADASIL patient show loss of function of *HTRA1* [24]. Mural cells induced from a hiPSC line that is derived from a patient with CADASIL exhibit co-localization of HTRA1 and mutant NOTCH3 extracellular domain [25]. In fact, in terms of signal transduction pathways, HTRA1 cleaves the NOTCH3 ligand JAG1, suggesting a close link between HTRA1 and Notch signaling. Given that *NOTCH3* expression was not altered at the iPSC level, further examination of *NOTCH3* expression or protein function would be informative to investigate whether *NOTCH3* is linked to the pathomechanisms of *HTRA1*-related CSVD using SM9-1 iPSC line differentiated into mural cells such as vascular smooth muscle cells or pericytes. Although our limitation is that we established a hiPSC line from only one donor patient due to rare and limited access to donors with identified mutations, our findings expand the possibility of studying the molecular pathomechanisms of CSVD using the patient-derived iPSC line.

## Conclusions

We established a hiPSC line derived from a female patient carrying a heterozygous R302Q mutation in *HTRA1* with clinical manifestations of CSVD such as stroke, dementia, multiple lacunar infarcts, and cervical spondylosis. In the generated hiPSC line, the causative gene *HTRA1* and its associated gene *NOG* showed altered expression levels. Since it is the first reported hiPSC line established from a patient with heterozygous *HTRA1*-related CSVD, it will be a valuable resource to elucidate a series of pathophysiology caused by the *HTRA1* mutation and the resultant decrease in HTRA1 protease activity. The disease modeling, including arteriopathy and spondylosis from the patient-derived iPSCs, would also contribute to the search for effective therapeutic targets and drugs.

## Methods

### Generation of iPSC and cell culture

Patient-derived PBMCs were isolated using BD Vacutainer CPT Mononuclear Cell Preparation Tube (BD Biosciences, NJ, USA). For T lymphocytes expansion and activation, the cells were cultured for 7 days with KBM502 (Kohjin Bio, Saitama, Japan) with Dynabeads Human T-Activator CD3/CD28 (Thermo Fisher Scientific, MA, USA) at 37 °C in 5% CO_2_. 3 ×10^6^ T lymphocytes were electroporated with pCE-hOCT3/4, pCE- hSK, pCE-hUL, pCE-mp53DD (0.63 μg each), and 0.5 μg pCXB-EBNA1 (Addgene #41813, 41814, 41855, 41856, and 41857) using Amaxa Human T cell Nucleofector Kit and Nucleofector 2b (Lonza, Basel, Switzerland) using the program: V-024 for PBMCs. Transfection was performed according to the manufacturers’ instructions. The transfected cells were seeded in an iMatrix 511 (Laminin511E8; Nippi, Tokyo, Japan)-coated 6-well plate in KBM502 (Kohjin Bio, Saitama, Japan) (set as day 0). StemFit AK02N medium (Ajinomoto, Tokyo, Japan) was added on days 3, 5, and 7. The medium was fully changed on day 9 and then every 3 days. After the emergence of iPSC colonies, they were mechanically isolated and respectively stocked in STEM-CELLBANKER GMP (Takara Bio, Shiga, Japan) after brief expansion. iPSCs were maintained in StemFit AK02N medium. iPSCs were passaged every 7 days. For passaging, after treating with 0.5 × TrypLE select (Thermo Fisher Scientific, MA, USA) diluted with PBS for 5 min at 37°C in 5% CO_2,_ the cells were dissociated and seeded at a density of 1.3 × 10^4^ in an iMatrix 511-coated 6-well plate. 10 μM Y27632 (Nacalai tesque, Kyoto, Japan) was supplemented on the first day to improve cell viability. The medium was changed on days 1, 3, and 5 from passaging. Feeder-free iPSCs were used and analyzed for alkaline phosphatase staining, immunocytochemistry, karyotyping, and mycoplasma contamination test.

For on-feeder culture conditions, iPSCs were seeded on mitotically inactivated SNL 76/7 feeder cells (Sigma, NY, USA) in hiPSC medium, which consists of D-MEM/Ham’s F-12 medium (Wako, Osaka, Japan) supplemented with 20% Knockout Serum Replacement (KSR) (Thermo Fisher Scientific, MA, USA), 2 mM L-glutamine, 0.8% Non-Essential Amino Acids (NEAA) (Sigma, NY, USA), 0.5% Penicillin/Streptomycin (Nacalai tesque, Kyoto, Japan), 10 ng/mL hbFGF (PeproTech, New Jersey, USA), and 0.1 mM 2-mercaptoethanol (Sigma, NY, USA). The medium was changed every day. The cells were detached with T-solution consisting of 25% trypsin, 100 mg/mL collagenase IV (Thermo Fisher Scientific, MA, USA), 1 mM CaCl_2_, and 20% KSR, and picked up for EBs formation.

### Alkaline phosphatase staining

Cells were fixed with 99.5% ethanol for 10 min at room temperature and washed 3 times with phosphate-buffered saline (PBS). Then, the cells were incubated with Alkaline phosphatase staining mixture B5655 (Sigma, NY, USA) for 20 min. The stained cells were washed 3 times with PBS and imaged using ECLIPSE TS100 microscope with DS-L3 system (Nikon, Tokyo, Japan).

### Detecting the expression of reprogramming factors

DNA was extracted using KAPA Mouse Genotyping Kit (Kapa Biosystems, MA, USA). PCR was performed with the following setting: 3 min at 95 °C; 35 cycles of 15 s at 95 °C, 15 s at 60 °C, and 15 s at 72 °C; incubation at 4 °C. PCR was conducted by ProFlex PCR System (Thermo Fisher Scientific, MA, USA) using KAPA Taq 2x ReadyMix (Kapa Biosystems, MA, USA). We used a 2% agarose gel for electrophoresis with 1 kb Plus DNA Ladder (Thermo Fisher Scientific, MA, USA). Primers are described in Supplementary Table S3.

### RNA extraction and RT-qPCR analysis

RNA was extracted using the RNeasy Micro Kit (Qiagen, Venlo, Netherlands), and cDNA was synthesized using iScript cDNA Synthesis Kit with DNase I treatment (Bio-Rad, CA, USA), which were performed according to manufacturers’ instructions. 4 ng of cDNA diluted in water was amplified with 20 μM forward and reverse primers (summarized in Supplementary Table S3), TB Green II, and ROX Dye II (Takara Bio, Shiga, Japan) for quantitative PCR by ViiA7 Real-Time PCR system (Thermo Fisher Scientific, MA, USA). The results were normalized by housekeeping *GAPDH* expression levels. The comparative Ct method (∆∆Ct method) was used for quantification. The expression levels of *OCT3/4* and *NANOG* were compared with healthy T lymphocytes. The reported iPS lines 201B7, WD39, and 414C2 were used as healthy controls [16, 26, 27].

### Immunocytochemistry

Cells were fixed with 4% paraformaldehyde for 20 min at room temperature and permeabilized and blocked with 1% fetal bovine serum (Sigma, NY, USA) and 0.3% Triton X-100 in PBS for 1 h at room temperature. The cells were incubated overnight at 4 °C with primary antibodies (summarized in Supplementary Table S3). Then, the cells were washed 3 times with PBS and incubated for 1 h with secondary antibodies. We used Alexa 488/555/647-conjugated secondary antibodies (1:1000, Thermo Fisher Scientific, MA, USA) and Hoechst 33342 (10 μg/ml, Merck, Darmstadt, Germany) for nuclear DNA staining in blue. The cells were then washed 3 times with PBS and imaged using the BZ-X810 microscope (Keyence, Osaka, Japan).

### DNA extraction and sequencing

For DNA extraction, cells were lysed in cell lysis buffer containing 0.2 M Tris-HCl, 10 mM EDTA, 0.2% SDS, 0.2 M NaCl, and 10 μg/ml proteinase K in nuclease-free water overnight at 55 °C. DNA was purified with a standard molecular biological method using TE-saturated phenol, 120 mM sodium acetate, and 70% ethanol, then solved and stored in TE buffer. The target region was amplified with the following setting: 30 s at 95 °C; 35 cycles of 10 s at 98 °C and 2 min at 68 °C; 10 min at 68 ° C; incubation at 4 °C. PCR was conducted by ProFlex PCR System using PrimeSTAR Max DNA polymerase (Takara Bio, Shiga, Japan). After electrophoresis using a 1% agarose gel, target DNA was extracted from the gel. Purified DNA amplicon was mixed with sequencing primers (summarized in Supplementary Table S3), and then Sanger sequencing was performed by Eurofins Genomics (Tokyo, Japan).

### In vitro spontaneous differentiation

On-feeder iPSCs cultured on mitotically inactivated SNL 76/7 feeder cells were transferred to Petri dishes in D-MEM/Ham’s F-12 medium containing 20% KSR, 2 mM L-glutamine, 0.1 M NEAA, 0.1 M 2-mercaptoethanol, and 0.5% Penicillin/Streptomycin. The medium was changed every 3 days. After culturing for 8 days in a floating state, the cells were attached to gelatin-coated plates in D-MEM/Ham’s F-12 medium supplemented with 10% fetal bovine serum. Additional culture duration is 15 days (for staining mesoderm and endoderm markers) and 29 days (for staining an ectoderm marker), respectively.

### Karyotyping and STR analysis

Karyotyping analysis was performed by LSI Medience Corporation (Tokyo, Japan). Twenty cells were randomly analyzed for G-banding. STR analysis was performed by Takara Bio (Shiga, Japan) using the extracted genome DNA from T lymphocytes and iPSCs.

### Mycoplasma detection assay

Mycoplasma contamination test was performed using LB960 microplate luminometer Centro (Berthold Technologies, Bad Wildbad, Germany). We used MycoAlert Mycoplasma Detection Kit and MycoAlert Assay Control Set (Lonza, Basel, Switzerland).

### Statistics

Values of RT-qPCR are the mean and geometric SEM of technical triplicates. Statistical significance was determined by Student’s *t* test. *P* values < 0.01 are represented by **; *p* values < 0.001 are represented by ***, and are considered statistically significant.

## Supplementary Information

**Additional file 1: Supplementary Table S1.** STR analysis. **Table S2.** Mycoplasma contamination analysis.

**Additional file 2: Supplementary Figure S1.** RT-qPCR analysis of pluripotency marker genes among iPSC lines.

**Additional file 3: Supplementary Table S3.** Reagent information.

## Data Availability

The authors confirm that the data supporting the findings of this study are available within the article and its supplementary materials.

## Abbreviations

CADASIL: Cerebral autosomal dominant arteriopathy with subcortical infarcts and leukoencephalopathy
CARASIL: Cerebral autosomal recessive arteriopathy with subcortical infarcts and leukoencephalopathy
CSVD: Cerebral small vessel disease
EBs: Embryoid bodies
FLAIR: Fluid attenuated inversion recovery
hiPSCs: Human induced pluripotent stem cells
HTRA1: High-temperature requirement serine peptidase A
KSR: Knockout serum replacement
mp53DD: Murine dominant-negative mutant of p53
MRA: Magnetic resonance angiography
MRI: Magnetic resonance image
NEAA: Non-essential amino acids
PBMCs: Peripheral blood mononuclear cells
RT-qPCR: Reverse transcription-quantitative real-time PCR
STR: Short tandem repeat
TGF-β1: Transforming growth factor β1
T2*WI: T2*-weighted image
