# Supplementary material for: A human induced pluripotent stem cell model from a patient with hereditary cerebral small vessel disease carrying a heterozygous R302Q mutation in HTRA1
Source: Inflamm Regen. 2023 Apr 3;43:23. doi: 10.1186/s41232-023-00273-7 (PMC10069112; doi:10.1186/s41232-023-00273-7)
Supplement: Supplementary file 1 — Additional file 1: Supplementary Table S1. STR analysis. Table S2. Mycoplasma contamination analysis. [file 41232_2023_273_MOESM1_ESM.doc]

**Supplementary information**

**Additional file 1:**

**Supplementary Table S1. STR analysis**

10 loci (TH01, D21S11, D5S818, D13S317, D7S820, D16S539, CSF1PO, AMEL, vWA, and TPOX) were tested and matched. The result shows that SM9-1 T cell and iPSC are derived from an identical female.

**Table S2. Mycoplasma contamination analysis**

The result of SM9-1 iPSC (0.353…) was lower than 0.9, which shows SM9-1 iPSC was negative for mycoplasma contamination.
