# Supplementary material for: A human induced pluripotent stem cell model from a patient with hereditary cerebral small vessel disease carrying a heterozygous R302Q mutation in HTRA1
Source: Inflamm Regen. 2023 Apr 3;43:23. doi: 10.1186/s41232-023-00273-7 (PMC10069112; doi:10.1186/s41232-023-00273-7)
Supplement: Supplementary file 2 — Additional file 2: Supplementary Figure S1. RT-qPCR analysis of pluripotency marker genes among iPSC lines. [file 41232_2023_273_MOESM2_ESM.doc]

**Supplementary information**

**Additional file 2:**

**Supplementary Figure S1. RT-qPCR analysis of pluripotency marker genes among iPSC lines**

The values represent the mean ± SEM of technical triplicates. There were no significant differences in the expression of *NANOG* and *OCT3/4* between healthy iPSC lines and SM9-1 iPSC line.
