# Supplementary material for: A human induced pluripotent stem cell model from a patient with hereditary cerebral small vessel disease carrying a heterozygous R302Q mutation in HTRA1
Source: Inflamm Regen. 2023 Apr 3;43:23. doi: 10.1186/s41232-023-00273-7 (PMC10069112; doi:10.1186/s41232-023-00273-7)
Supplement: Supplementary file 3 — Additional file 3: Supplementary Table S3. Reagent information. [file 41232_2023_273_MOESM3_ESM.doc]

**Supplementary information**

**Additional file 2:**

**Supplementary Table S3. Reagent information**

| **Antibodies used for immunocytochemistry** | | | |
| --- | --- | --- | --- |
| **Markers** | **Antibody** | **Dilution** | **Company Cat # and RRID** |
| Pluripotency Markers | Mouse anti-TRA-1-60 | 1:500 | Cell Signaling Technology, Cat# 4746, RRID:AB_ 2119059 |
| Pluripotency Markers | Mouse anti-TRA-1-81 | 1:500 | Cell Signaling Technology, Cat# 4745, RRID:AB_ 2119060 |
| Pluripotency Markers | Rat anti-SSEA3 | 1:500 | Abcam, Cat# ab16286, RRID:AB_882700 |
| Pluripotency Markers | Mouse anti-OCT3/4 | 1:500 | Santa Cruz, Cat# sc-5279, RRID:AB_628051 |
| Pluripotency Markers | Rabbit anti-NANOG | 1:250 | Cosmo Bio, Cat# REC-RCAB0003P, RRID:AB_1962353 |
| Differentiation Markers | Rat anti-MSI1 | 1:500 | Thermo Fisher Scientific, Cat# 14-9896-82, AB_10598804 |
| Differentiation Markers | Rabbit anti-FOXA2 | 1:500 | Cell Signaling, Cat# 8186, AB_10891055 |
| Differentiation Markers | Mouse anti-αSMA | 1:300 | Dako Cat# M0851, AB_2223500 |
| **Primers** | | | |
|  | **Target** | **Amplicon Size** | **Forward/Reverse primer (5′-3′)** |
| Housekeeping gene (RT-qPCR) | *GAPDH* | 138 bp | GCACCGTCAAGGCTGAGAAC  TGGTGAAGACGCCAGTGGA |
| Pluripotency Markers  (RT-qPCR) | *NANOG* | 154 bp | TGAACCTCAGCTACAAACAG  TGGTGGTAGGAAGAGTAAAG |
| Pluripotency Markers (RT-qPCR) | *OCT3/4* | 144 bp | GACAGGGGGAGGGGAGGAGCTAG  CTTCCCTCCAACCAGTTGCCCCA |
| Phenotyping (RT-qPCR) | *HTRA1* | 119 bp | TCCCAACAGTTTGCGCCATAA  CCGGCACCTCTCGTTTAGAAA |
| Phenotyping (RT-qPCR) | *HTRA1* mutant allele | 209 bp | AAGCCAAAATCAAGGATGTGGAT  CCCCAGCTCTTTGCCGCCTT |
| Phenotyping (RT-qPCR) | *HTRA1* wild-type allele | 183 bp | AGAAAGCAGACATCGCACTCATC  CCAGCTCTTTGCCGCCTC |
| Phenotyping (RT-qPCR) | *NOG* | 337 bp | CCATGCCGAGCGAGATCAAA  TCGGAAATGATGGGGTACTGG |
| Phenotyping (RT-qPCR) | *NOTCH3* | 122 bp | CGTGGCTTCTTTCTACTGTGC  CGTTCACCGGATTTGTGTCAC |
| Pluripotency Markers (PCR) | Endogenous *OCT3/4* | 113 bp | AGTTTGTGCCAGGGTTTTTG  ACTTCACCTTCCCTCCAACC |
| Episomal Plasmids (PCR) | oriP plasmid | 544 bp | TTCCACGAGGGTAGTGAACC  TCGGGGGTGTTAGAGACAAC |
| Episomal Plasmids (PCR) | *EBNA1* plasmid | 667 bp | ATCGTCAAAGCTGCACACAG  CCCAGGAGTCCCAGTAGTCA |
| Targeted mutation analysis/sequencing | *HTRA1* exon4 | 486 bp | AGTTACCTCCCCACGGTTTC  CACCCGCAGAGGGACAGAG |
